# A 14-gene gemcitabine resistance gene signature is significantly associated with the prognosis of pancreatic cancer patients

**DOI:** 10.1038/s41598-021-85680-x

**Published:** 2021-03-17

**Authors:** Xing Wei, Xiaochong Zhou, Yun Zhao, Yang He, Zhen Weng, Chunfang Xu

**Affiliations:** 1 Department of Gastroenterology, The First Affiliated Hospital of Soochow University, Suzhou, 215006 China; 2 MOE Engineering Center of Hematological Disease, Soochow University, Suzhou, 215123 China; 3 Cyrus Tang Hematology Center, Soochow University, Suzhou, 215123 China; 4 National Clinical Research Center for Hematologic Diseases, The First Affiliated Hospital of Soochow University, Suzhou, 215006 China; 5 MOH Key Lab of Thrombosis and Hemostasis, Jiangsu Institute of Hematology, The First Affiliated Hospital of Soochow University, Suzhou, 215006 China; 6 Collaborative Innovation Center of Hematology, Soochow University, Suzhou, 215006 China

**Keywords:** Cancer, Data integration, Data mining, Data processing

## Abstract

To identify a gemcitabine resistance-associated gene signature for risk stratification and prognosis prediction in pancreatic cancer. Pearson correlation analysis was performed with gemcitabine half maximal inhibitory concentration (IC50) data of 17 primary pancreatic cancer lines from Genomics of Drug Sensitivity in Cancer (GDSC) and the transcriptomic data from GDSC and Broad Institute Cancer Cell Line Encyclopedia, followed by risk stratification, expression evaluation, overall survival (OS) prediction, clinical data validation and nomogram establishment. Our biomarker discovery effort identified a 14-gene signature, most of which featured differential expression. The 14-gene signature was associated with poor OS in E-MTAB-6134 (HR 2.37; 95% CI 1.75–3.2; *p* < 0.0001), pancreatic cancer-Canada (PACA-CA) (HR 1.76; 95% CI 1.31–2.37; *p* = 0.00015), and 4 other independent validation cohorts: pancreatic cancer-Australia (PACA-AU) (HR 1.9; 95% CI 1.38–2.61; *p* < 0.0001), The Cancer Genome Atlas (TCGA) (HR 1.73; 95% CI 1.11–2.69; *p* = 0.014), GSE85916 (HR 1.97; 95% CI 1.14–3.42; *p* = 0.014) and GSE62452 (HR 1.82; 95% CI 1.02–3.24; *p* = 0.039). Multivariate analysis revealed that the 14-gene risk score was an independent pancreatic cancer outcome predictor in E-MTAB-6134 (*p* < 0.001) and TCGA (*p* = 0.006). A nomogram including the 14-gene was established for eventual clinical translation. We identified a novel gemcitabine resistance gene signature for risk stratification and robust categorization of pancreatic cancer patients with poor prognosis.

## Introduction

Pancreatic cancer is one of the most dangerous malignancies worldwide, with a 5-year overall survival of less than 5% and an approximately 6–8-month median survival after diagnosis^1,2^. Surgical resection followed by adjuvant treatment with the nucleoside analog drug gemcitabine is the standard management of pancreatic cancer patients in clinical practice^3^. Moreover, nab-paclitaxel plus gemcitabine (Nab-Gem) and FOLFIRINOX (folinic acid, 5-fluorouracil, irinotecan and oxaliplatin) represent the standard regimens for the first-line treatment of locally advanced and metastatic pancreatic cancer^4,5^. However, the majority of patients treated with gemcitabine chemotherapy eventually develop gemcitabine resistance^6^. Obviously, the effectiveness of gemcitabine chemotherapy is related to the prognosis of pancreatic cancer and therefore could be employed as a baseline for treatment and prognosis improvement of this fatal disease.

Many studies have been carried out to elucidate the possible mechanisms involved in gemcitabine resistance^7–9^, which are considered to be related to transport and metabolism behavior and are thought to involve multiple enzymes and signaling pathways, including human concentrative nucleoside transporters (hCNTs), human equilibrative nucleoside transporters (hENTs), deoxycytidine kinase (dCK), and ribonucleotide reductase (RR), or increased activity of the detoxifying enzyme cytidine deaminase (CDA), and PI3K/Akt, MAPK and NF-κB pathways. Based on these findings, changes in multiple genes at the functional and expression levels could be a reasonable phenomenon during the development of gemcitabine resistance. Recent advances in microarray gene chip technology and high-throughput sequencing have resulted in protocols for evaluating the expression level changes of multiple genes at the same time. Moreover, the generation of large public databases with abundant clinicopathological information and molecular profiling data, including genomic, transcriptomic and epigenomic data, of multiple different types of cancer has facilitated cancer research. In particular, The Cancer Genome Atlas (TCGA)^10^ with over 20,000 primary cancer and matched normal samples from 33 cancer types, the International Cancer Genome Consortium^11^ (ICGC, containing Pancreatic Cancer-Canada [PACA-CA], and Pancreatic Cancer-Australia [PACA-AU]) with 50 different cancer types, and the international functional genomics public data repositories Gene Expression Omnibus (GEO) and ArrayExpress^12^ are the most commonly accessed repositories. In addition, drug activity information for approximately 1400 cell lines is available from the Genomics of Drug Sensitivity in Cancer (GDSC)^13^ and the Broad Institute Cancer Cell Line Encyclopedia (CCLE)^14^. However, although high-throughput-sequencing-based studies have revealed several prognosis-related gene signatures based on differentially expressed mRNAs for predicting overall survival (OS) in pancreatic cancer^15–17^, few studies have employed a gemcitabine resistance-related gene signature for those purposes in pancreatic cancer (according to our literature search results).

In the present study, by including genes related to gemcitabine resistance in pancreatic cancer, we performed a systematic and comprehensive discovery and validation of biomarkers to identify and generate a gene expression signature for the effective prognostic prediction of patients with pancreatic cancer by using multiple datasets. We identified a novel 14-gene signature comprising genes related to gemcitabine resistance, which could offer excellent accuracy for patient risk stratification and prognosis prediction.

## Results

### Identification of the gemcitabine resistance- and survival-related gene signature, establishment of the prognostic signature and evaluation of the expression of the identified genes

**Figure 1:**
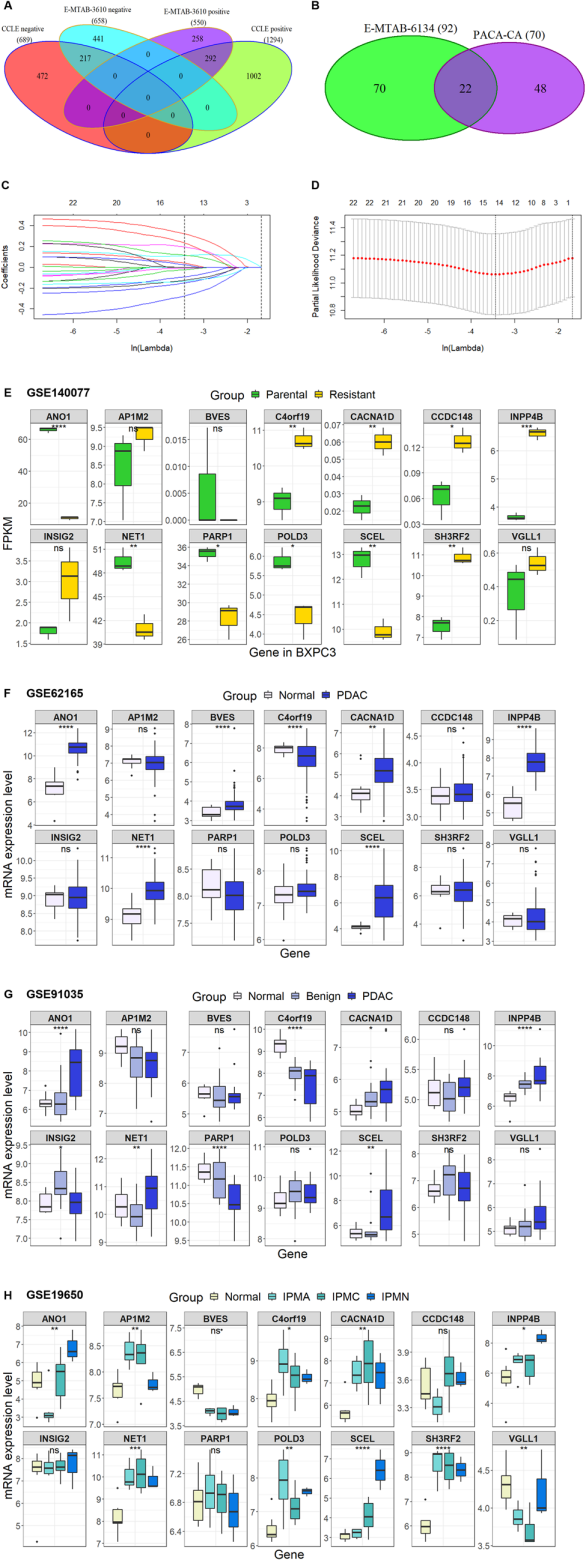
Identification of 14-gene signature and evaluation of the gene expression in multiple GEO datasets with different cell and clinical samples. (**A**) Venn diagram of the positive and negative gemcitabine resistance correlated genes derived from E-MTAB-3610 and CCLE datasets. (**B**) Venn diagram of identification of 22 survival related genes by Univariate Cox analysis via using E-MTAB-6134 and PACA-CA containing above gemcitabine resistance correlated genes and the clinical data. (**C**,**D**) Fourteen genes were identified by Lasso Cox regression analysis; Lasso coefficients profiles (**C**) and Lasso deviation profiles (**D**). Gene expression level evaluation of the 14 genes in GSE140077 (gemcitabine resistance cells; FPKM: Fragments Per Kilobase of transcript per Million mapped reads; (**E**), GSE62165 (PDAC and normal tissues; (**F**), GSE91035 (PDAC, benign and normal tissues; (**G**) and GSE19650 (multiple different pre-pancreatic carcinoma stage tissues; IPMA: intraductal papillary-mucinous adenoma; IPMC: intraductal papillary-mucinous carcinoma; IPMN: intraductal papillary-mucinous neoplasm; (**H**).

A total of 1208 (550 positively correlated and 658 negatively correlated genes) and 1983 (1294 positively correlated and 689 negatively correlated genes) gemcitabine resistance-related genes were identified from the E-MTAB-3610 and CCLE datasets, respectively (Fig. 1A). The intersection of these two datasets revealed 509 genes (292 positively correlated and 217 negatively correlated). After combination analysis of the clinical data from E-MTAB-6134 and PACA-CA, 92 and 70 survival-related genes were obtained, respectively, and 22 intersecting genes were found (Fig. 1B). Finally, the efficacy of a 14-gene signature was calculated by Lasso Cox regression (Fig. 1C,D), and the detailed gene screening data of each step are shown in the Supplementary file. These genes included CCDC148, SH3RF2, CACNA1D, POLD3, PARP1, AP1M2, C4orf19, ANO1, VGLL1, SCEL, INPP4B, NET1, INSIG2 and BVES, and 6 of these genes were reported by previous studies (detailed shown in the discussion). We then established the following equation: Risk score = − 0.2750 × CCDC148 − 0.1514 × SH3RF2 − 0.1332 × CACNA1D − 0.1250 × POLD3-0.0932 × PARP1 − 0.0244 × AP2 − 0.0105 × C4orf19 + 0.0238 × ANO1 + 0.0387 × VGLL1 + 0.11 × SCEL + 0.1158 × INPP4B + 0.1943 × NET1 + 0.2316 × INSIG2 + 0.3211 × BVES (Table 1). Next, we evaluated the expression profiles of these 14 genes in 4 different Gene Expression Omnibus (GEO) datasets, and out of the 14, the numbers of differentially expressed genes in the GSE140077 (gemcitabine-resistant cells; Fig. 1E), GSE62165 (PDAC and normal tissues; Fig. 1F), GSE91035 (PDAC, benign and normal tissues; Fig. 1G) and GSE19650 (multiple different pre-pancreatic carcinoma stage tissues; Fig. 1H) datasets were 10, 7, 7 and 10, respectively (Table 2), suggesting that most of these genes had abnormal expression in pancreatic cancer.

**Table 1:** Detailed information of the correlated genes.

Gene symbol	Coefficient number	Gene function
CCDC148	− 0.275	Diseases associated include Tyrosinemia, Type Ii
SH3RF2	− 0.1514	Gene Ontology (GO) annotations include *ligase activity* and *protein phosphatase inhibitor activity*
CACNA1D	− 0.1332	GO annotations include *ion channel activity* and *ankyrin binding*
POLD3	− 0.125	GO annotations include *DNA-directed DNA polymerase activity*
PARP1	− 0.0932	GO annotations include protein kinase binding
AP1M2	− 0.0244	Among its related pathways are Clathrin derived vesicle budding and Nef-mediates down modulation of cell surface receptors by recruiting them to clathrin adapters
C4orf19	− 0.0105	NA
ANO1	0.0238	GO annotations include *protein homodimerization activity and intracellular calcium activated chloride channel activity*
VGLL1	0.0387	GO annotations include *transcription coactivator activity*
SCEL	0.11	Diseases associated with SCEL include Extrahepatic Bile Duct Adenocarcinoma
INPP4B	0.1158	GO annotations include *lipid binding and phosphatidylinositol trisphosphate phosphatase activity*
NET1	0.1943	GO annotations include guanyl-nucleotide exchange factor activity and GTP-Rho binding
INSIG2	0.2316	GO annotations include *transcription factor binding*
BVES	0.3211	GO annotations include *structural molecule activity and cAMP binding*

*NA* Not available.

**Table 2:** Detailed information of the expression pattern of the correlated genes.

Genes	Downregulated genes	Upregulated genes	Total numbers
14-gene in GSE140077	ANO1, NET1, PARP1, POLD3, SCEL,	C4orf19, CACNA1D, CCDC148, INPP4B, SH3RF2	10
14-gene in GSE62165	C4orf19	ANO1, BVES, CACNA1D, INPP4B, NET1, SCEL	7
14-gene in GSE91035	C4orf19, PARP1	ANO1, CACNA1D, INPP4B, NET1, SCEL	7
14-gene in GSE19650	VGLL1	ANO1, C4orf19, CACNA1D, INPP4B, NET1, SCEL, SH3RF2	10*

*Expression pattern is not consistent in different type of pancreatic cancer. The total number represents the genes differential expressed pattern.

### Identification of a 14-gene risk stratification signature for the prognosis of patients with pancreatic cancer and exploration of the expression pattern

**Figure 2:**
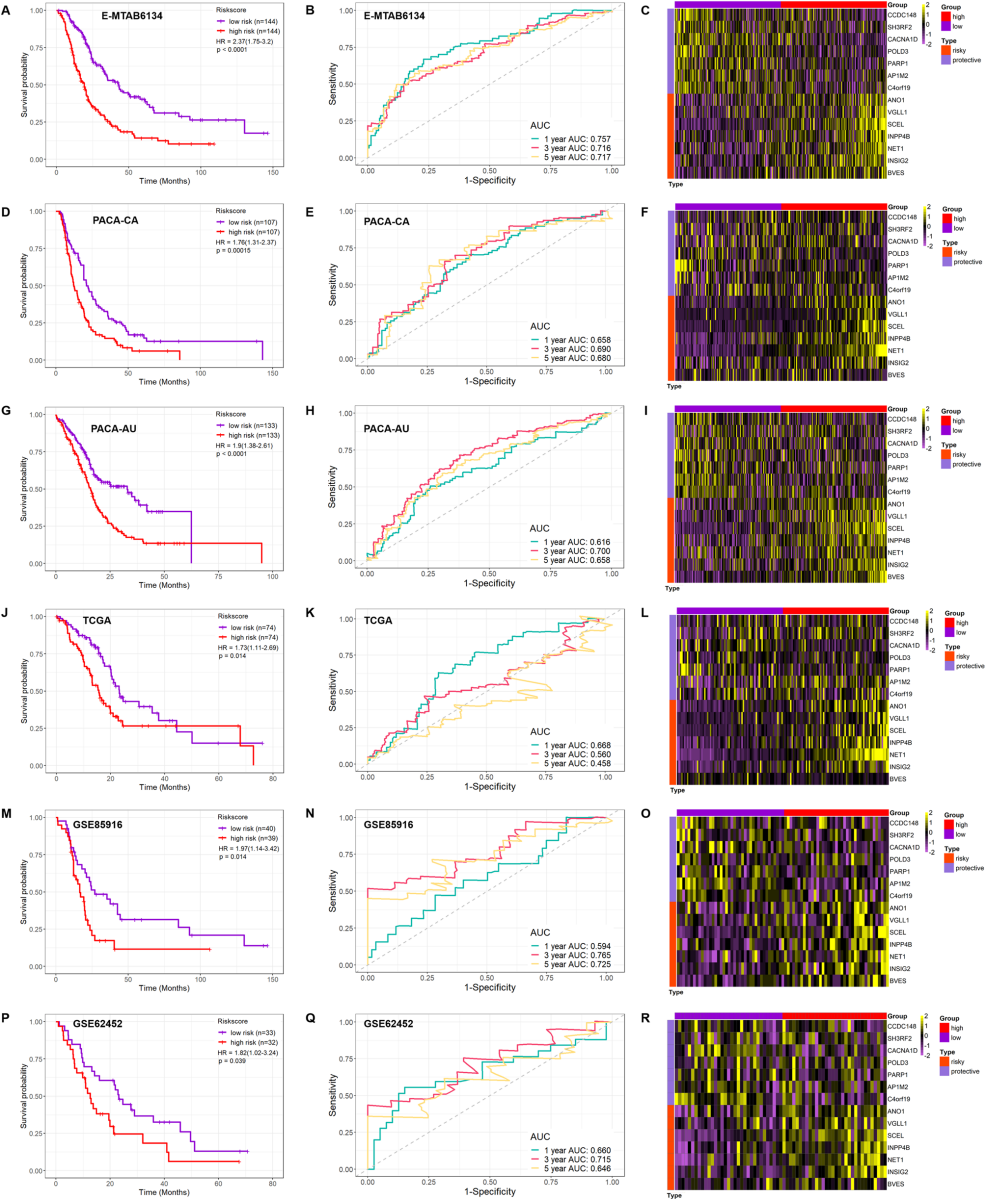
Identification of 14-gene risk stratification signature for prognosis of patients with pancreatic cancer and exploration of the expression pattern. Kaplan–Meier curve, receiver operator characteristic curves for 1, 3 and 5-year overall survival prediction and 14 genes expression heatmaps of different datasets were shown. (**A**–**C**) E-MTAB-6134; (**D**–**F**) PACA-CA; (**G**–**I**) PACA-AU; (**J**–**L**) TCGA; (**M**–**O**) GSE85916; (**P**–**R**) GSE62452.

Subsequently, we used multiple datasets to validate the 14-gene signature. The results of Kaplan–Meier curve uncovered significantly favorable overall survival in patients with a low risk score from the E-MTAB-6134 (HR 2.37; 95% CI 1.75–3.2; *p* < 0.0001), PACA-CA (HR 1.76; 95% CI 1.31–2.37; *p* = 0.00015), PACA-AU (HR 1.9; 95%CI 1.38–2.61; *p* < 0.0001), TCGA (HR 1.73; 95% CI 1.11–2.69; *p* = 0.014), GSE85916 (HR 1.97; 95% CI 1.14–3.42; *p* = 0.014) and GSE62452 (HR 1.82; 95% CI 1.02–3.24; *p* = 0.039) (Fig. 2A,D,G,J,M,P) datasets but not in the GSE71729 dataset (HR 0.98; 95% CI 0.63–1.51; Supplementary Fig. 1). Moreover, the prediction efficacy of the 14-gene signature was also evaluated by ROC curves; in most of the datasets, the AUCs for 1-, 3- and 5-year OS was larger than 0.6 and close to 0.7. The highest AUC was found in the E-MTAB-6134 set, and the AUCs for 1-, 3- and 5-year OS were 0.757, 0.716 and 0.717, respectively, while the AUCs for 3- and 5-year OS were relatively low in the TCGA set (0.560 and 0.458, respectively) (Fig. 2B,E,H,K,N,Q and Supplementary Fig. 1). Detailed information on the risk score and survival status can be found in Supplementary Fig. 2. In addition, we evaluated the expression pattern of these 14 genes in the above datasets, and the heatmap results showed a pattern of differential expression of these genes in patients with high and low risk scores (Fig. 2C,F,I,L,O,R and Supplementary Fig. 1). These results suggested that the 14-gene signature could be effectively employed for predicting overall survival and that the differentially expressed pattern of these 14 genes could be found in most of the patients.

### Identification of the 14-gene signature as an independent risk and survival outcome predictor in pancreatic cancer patients

**Figure 3:**
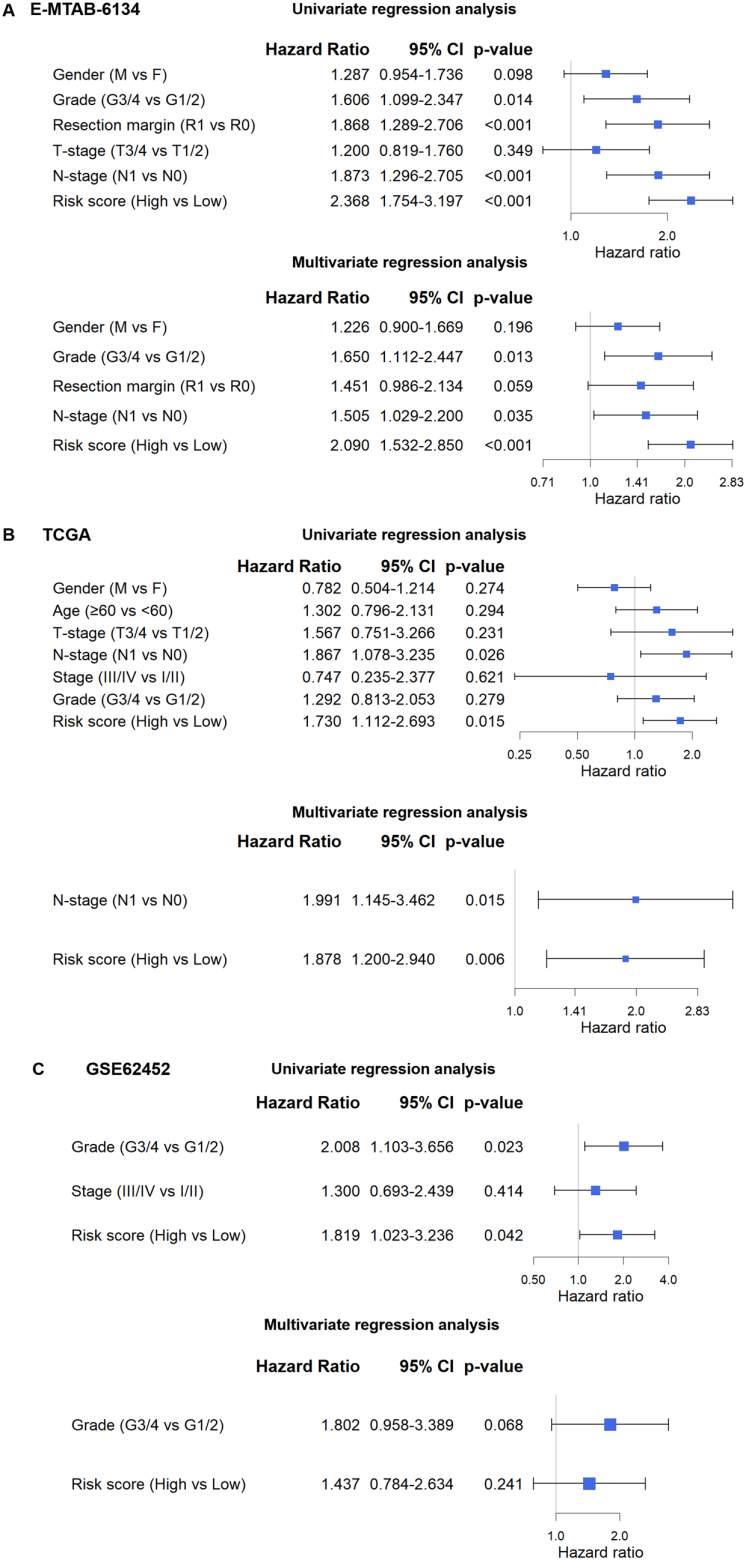
Performance of 14-gene signature as a predictor of risk and survival outcomes in pancreatic cancer patients by univariate and multivariate Cox analysis including clinicopathological features. (**A**) E-MTAB-3164; (**B**) TCGA; (**C**) GSE62452.

We then performed univariate and multivariate Cox regression analyses on this gene panel by including various clinicopathological features of pancreatic cancer patients in 3 datasets with available clinical information. In the univariate analysis, the 14-gene risk score was identified as a significant predictor of overall survival in all 5 datasets (E-MTAB-6134: *p* < 0.001; TCGA: *p* = 0.015; GSE62452: *p* = 0.042; PACA-AU: *p* < 0.001; PACA-CA: *p* < 0.001) (Fig. 3A–C and Supplementary Fig. 3). Further multivariate analysis revealed that the 14-gene risk score was an independent predictor in pancreatic cancer patients from the E-MTAB-6134 (*p* < 0.001) and TCGA (*p* = 0.006) datasets but not the GSE62452 dataset (*p* = 0.241) (Fig. 3A–C). Since there were relatively large numbers of patients in the E-MTAB-6134 and TCGA datasets, our results verified that our 14-gene signature is quite robust and can be used as an independent factor for survival prediction in pancreatic cancer patients.

### The 14-gene signature robustly identifies poor molecular subtypes in pancreatic cancer

**Figure 4:**
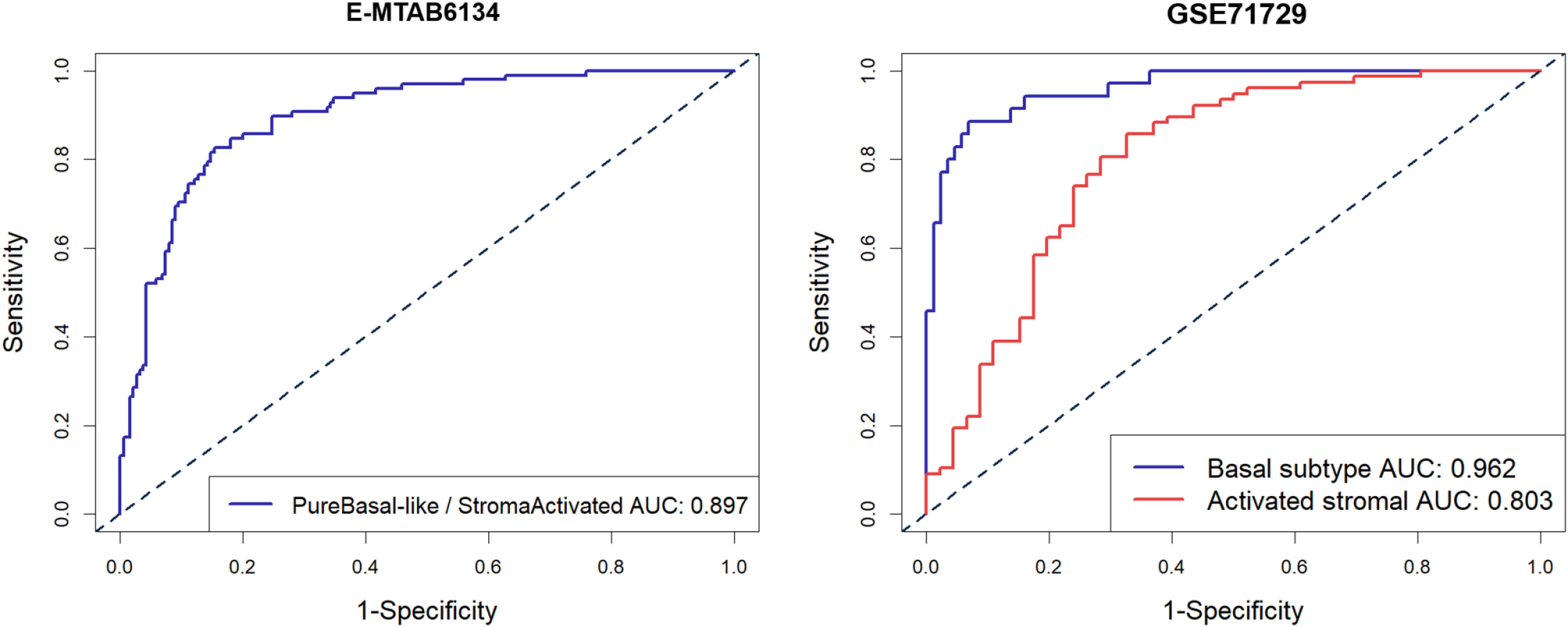
The 14-gene signature robustly identifies poor molecular subtypes in pancreatic cancer. Using the datasets E-MTAB-6134 and GSE71729, and a higher AUC value between 0.8 and 0.96 could be achieved by using the 14-gene signature.

Moreover, recently discovered transcriptomic data-based molecular subtypes, including the squamous subtype defined by Bailey^18^, the quasimesenchymal (QM) subtype by Collisson^19^, the basal-like subtype by Moffitt^20^, and the pure basal-like/stroma activated subtype by Puleo^21^, are predicted to have poor survival outcomes among PDAC patients. Due to the incomplete clinical information in some of the datasets, we only verified the effectiveness of our 14-gene signature for molecular subtype prediction in the E-MTAB-6134 and GSE71729 sets, and an AUC value between 0.8 and 0.96 could be achieved by using our signature (Fig. 4).

### Establishment of a risk nomogram for survival prediction in pancreatic cancer patients

**Figure 5:**
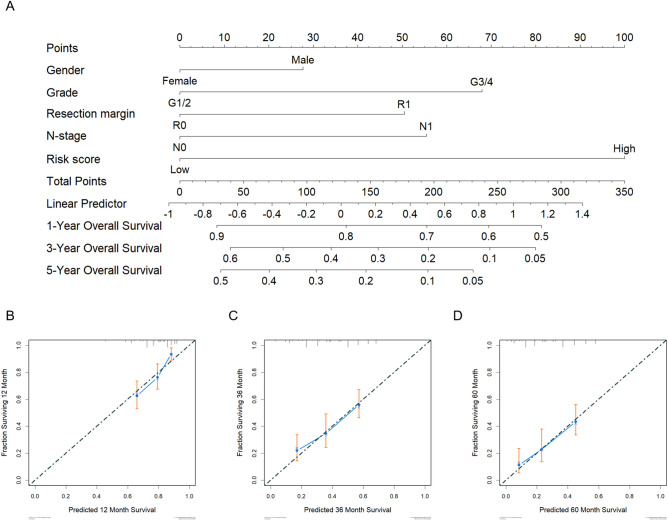
Nomograms Predicting Survival by 14-gene signature in pancreatic cancer patients. (**A**) The nomogram for 1-, 3- and 5-year overall survival prediction was established based on 4 independent prognostic factors; (**B**,**C**,**D**) Calibration plots for predicted and actual 1-, 3- and 5-year overall survival probabilities comparison. The 284-patient bootstrapped calibration plot for 1-, 3- and 5-year overall survival prediction is exhibited. The dotted line stands for the ideal fit; circles stand for nomogram-predicted probabilities; stars stand for the bootstrap-corrected estimates; and error bars stand for the 95% CIs of these estimates.

To further strengthen the accuracy of the predictive power of our 14-gene signature, we established a ready-to-use and clinically applicable risk nomogram for survival prediction in pancreatic cancer patients from E-MTAB-6134 to assess the performance of this signature via a combination of other univariate significant patient characteristic parameters (e.g., grade and N-stage). As shown in Fig. 5A, a higher total score calculated by adding the assigned numbers of each factor included in the nomogram was associated with worse 3-year and 5-year overall survival rates. For example, a patient with a higher grade and a higher 14-gene score could generate a total of 168 points (68 points for high grade and 100 points 14-gene high), with predicted 1-year, 3-year and 5-year overall survival rates of 73.0%, 25.0% and 13.0%, respectively. To validate the prediction model, the performance of the nomogram was evaluated by the discrimination index calculation and calibration plot adjusting for the 1-year, 3-year and 5-year survival. The accuracy of the predictive nomogram was higher than that of the 14-gene signature alone, with an accompanying C-statistic discriminatory index value of 0.67. This higher C-statistic revealed that the 14-gene signature in combination with sex, tumor grade, resection margin and N-stage was considerably robust in discriminating subjects with variable outcomes. The 284-patient bootstrapped calibration plots for the prediction of 1-year, 3-year and 5-year overall survival rates are shown in Fig. 5B–D. The calibration plots of the 14-gene signature with sex, grade, resection margin and N stage exhibited excellent consistency between the observed outcomes and predicted survival. These results further support the clinical significance of our 14-gene signature in combination with sex, grade, resection margin and N stage, which has superior overall predictive power for distinguishing survival outcomes in pancreatic cancer patients.

## Discussion

In addition to the application of conventional clinicopathological parameters or AJCC staging for prognosticating disease progression, molecular prognostic markers with the features of easy detection and simple quantification by standard methods could be employed not only for early diagnosis and prognosis prediction but also for novel therapeutic target discovery. Moreover, the combination of a panel of molecular biomarkers could overcome the hurdles resulting from tumor heterogeneity. In order to strengthen the survival and prognosis of pancreatic cancer patients, elucidate the mechanisms involved in gemcitabine resistance and discover novel diagnostic, prognostic and therapeutic-related biomarkers, we performed a preset study focusing on the relationship of gemcitabine resistance and overall survival. A novel 14-gene signature comprising CCDC148, SH3RF2, CACNA1D, POLD3, PARP1, AP1M2, C4orf19, ANO1, VGLL1, SCEL, INPP4B, NET1, INSIG2 and BVES and a corresponding risk score formula were established for pancreatic cancer risk stratification and prognosis prediction. Patients in the low-risk group had significantly better prognosis than those in the high-risk group. In addition, we confirmed the effectiveness of the 14-gene signature in predicting 1-year, 3-year and 5-year overall survival via AUC calculation.

As a widely employed prognosis evaluation modality in clinical oncology, nomograms can integrate different prognostic determinants, including molecular and clinicopathological characteristics^22^. The different probabilities of clinical events can be visualized and calculated into a total score for risk illustration. Compared to conventional staging methods, nomograms may more effectively strengthen clinical decision-making regarding prognosis prediction and thereby be beneficial for implementing timely treatment. The C-index and calibration curves identified that the 14-gene signature was effective for predicting 1-year, 3-year and 5-year overall survival. An integrated nomogram using the 14-gene signature and clinicopathological characteristics was set up for accurate overall survival prediction. As a supplement to AJCC staging and conventional clinicopathological characteristics, the 14-gene signature and nomogram could be employed as useful high-risk indicators and overall survival predictors.

With the advances in gene sequencing technologies, several groups have reported their own mRNA-level prognostic gene signatures that have the ability to predict overall survival in pancreatic cancer. Birnbaum et al. suggested a 25-gene signature based on clinicopathological parameters and gene expression data that predicts postoperative OS independent of classical factors and molecular subtypes^17^. Raman et al. reported a 5-gene prognostic model comprising ADM, ASPM, DCBLD2, E2F7, and KRT6A for accurate prediction of overall survival using the PAAD datasets from the TCGA^15^. Yan et al. confirmed that a 4-gene signature (CDC6, IGF2BP2, KNTC1, and LYRM) was significantly related to the progression and prognosis of pancreatic cancer^16^. Most recently, Wu et al. identified a nine-gene signature comprising ANKRD22, ARNTL2, CEP55, COL17A1, ITGB6, MCOLN3, MET, KLK10, and SLC25A45 for predicting overall survival of pancreatic cancer^23^. In addition, Kandimalla et al. identified a 15-gene immune, stromal and proliferation (ISP) gene signature comprising TNFRSF4, TNFSF18, TNFSF10, RFC4, PVRL3, PLD4, KDM6B, INHBA, IL32, IL4, IFIT3, FOXP3, CDC20, CD160, and AHR with favorable prediction of poor OS and identification of the molecular subtype of PDAC^24^. Considering that recent progress in combination therapies includes various chemotherapeutic regimens, especially the frequent use of gemcitabine in neoadjuvant and palliative therapies of pancreatic cancer patients, genes related to gemcitabine resistance could be a group of strong candidate genes in the identification of robust prognostic and predictive risk-stratification biomarkers for pancreatic cancer patients. Moreover, plenty of the available clinical and gene expression information from previous studies (including International Cancer Genome Consortium [ICGC], TCGA and GEO) could be used to validate the predictive power of these genes.

Among our panel of 14 genes included in the signature, 6 genes were previously studied in pancreatic cancers. PARP-1 plays critical roles in DNA damage repair during intrinsic cell death, and cytoplasmic PARP-1 was recently confirmed to promote pancreatic cancer tumorigenesis and resistance^25^. ANO1 located on amplicon 11q13 is usually amplified in human cancers with poor prognosis and is pivotal in PDAC cell migration^26^. SCEL is a potential biomarker for the prognosis of pancreatic cancer confirmed by Cheng et al^27^. INPP4B, which was first described by Zhai et al., is an oncogenic gene in pancreatic cancer and could serve as a potential diagnostic marker and an independent prognostic marker, suggesting that it is a novel therapeutic target for pancreatic cancer^28^. INSIG2 overexpression is related to the malignant phenotype of pancreatic cancer under hypoxic conditions^29^. BVES, as a novel regulator of the Rac1 and Cdc42 signaling cascades, controls cell shape and movement in multiple cancers, including pancreatic cancer^30^. In addition, CACNA1D was found to be expressed on pancreatic islets and play a vital role in insulin secretion, which is involved in β-cell physiology and pathophysiology^31^, and AP1M2 expression was found to be upregulated only in pancreatic cancer^32^. Other genes, such as CCDC148^33^, SH3RF2^34^, VGLL1^35^ and NET1^36^, were found to regulate different cell behaviors (such as cell proliferation and migration, cell death and cell cycle) involved in the development and progression of gastric cancer, whereas POLD3 was reported to be required for cell cycle progression and DNA synthesis and to be responsible for the high frequency of genomic duplications in human cancers^37^. No study was found on role of C4orf19 in cancers. Since most of the genes were previously reported to be involved in cancer biology, the findings of our panel are believable and could be useful. Moreover, since the 14 genes were selected using the Lasso regression method, the inconsistency in the expression patterns of the 14 genes at different levels (cell lines, normal vs. cancer, and specimens of different disease stages) could be neglected. In addition, we did not observe statistically significant correlations between the gemcitabine IC50 data and the mRNA expression levels of SPARC (*r* = − 0.444 and *p* = 0.074 in E-MTAB-3610, and *r* = − 0.503 and *p* = 0.056 in CCLE), hENT1 (*r* = − 0.162 and *p* = 0.535 in E-MTAB-3610, and *r* = − 0.450 and *p* = 0.093 in CCLE) and dCK (*r* = 0.303 and *p* = 0.236 in E-MTAB-3610, and *r* = 0.242 and *p* = 0.386 in CCLE), despite previous reports describing their roles in gemcitabine resistance^7–9^.

There are several limitations in this study. First, although datasets from several databases were employed, including the E-MTAB-6134, ICGC, TCGA and GEO databases, the main information on clinical characteristics and gene expression was from white, black or Hispanic populations mainly living in Europe and the United States; therefore, caution should be taken when applying the results to other ethnicities. Second, the establishment and verification of the nomogram were only performed using the E-MTAB-6134 database due to the lack of complete clinical information in other datasets. We did not include a clinical cohort of pancreatic cancer for validation due to failure to obtain enough clinical samples and related follow-up information in a relatively short study period. Third, missing clinical information (age, etc.) in some of the datasets (E-MTAB-6134, GSE85916, GSE62452) resulted in the inability to perform univariate and multivariate prognostic analyses. Fourth, the molecular mechanisms of the genes identified here and their roles in the pathogenesis and progression of pancreatic cancer require further experimental studies. Therefore, sample collection with complete experimental and clinical information should be performed for future validation.

In conclusion, we believe that the 14-gene signature obtained in the present study could provide an efficient platform for better managing patients with this devastating malignancy in clinical practice.

## Methods

### Acquisition of gene expression, half maximal inhibitory concentration (IC50) of gemcitabine and clinical data

Gene expression data and gemcitabine IC50 data of specific pancreatic cancer lines were obtained from GDSC (https://www.cancerrxgene.org/). Simultaneously, RNAseq data (CCLE_RNAseq_rsem_genes_tpm_20180929.txt.gz) and related annotation files (gencode.v19.genes.v7_model.patched_contigs.gtf.gz) were obtained from the CCLE (https://portals.broadinstitute.org/ccle).

The mRNA expression and related experimental and clinical data of pancreatic cancer samples were downloaded from GEO (http://www.ncbi.nlm.nih.gov/geo/) using the search terms “pancreatic cancer”, “gemcitabine resistance” and “expression profiling by array”. The gene expression microarray datasets GSE140077, GSE19650, GSE62165, GSE62452, GSE71729, GSE85916 and GSE91035 were selected and downloaded. The criteria for dataset selection were as follows: cell lines with gemcitabine resistance features or clinical samples with detailed clinical, survival and gene expression information. Among these datasets, GSE140077, GSE62165, GSE91035 and GSE19650 were used for validation of the expression level of the 14 identified gemcitabine resistance genes at the cellular and tissue levels, whereas clinical and gene expression data from GSE85916, GSE62452, and GSE71729 were used for validation of 14-gene signature in predicting prognosis. Detailed information on these microarray datasets is listed in Supplementary Table 1.

Microarray gene expression data from E-MTAB-6134 (N = 288; training cohort), transcriptomic data of PACA-CA (N = 214; validation cohort) and PACA-AU (N = 266; validation cohort) from ICGC and transcriptomic data from the TCGA (N = 148; validation cohort) were used for validation of 14-gene signature in predicting prognosis. The clinical and normalized ICGC RNA-sequencing data for PACA-CA and array-based gene expression data for PACA-AU were downloaded from the ICGC Data Portal (https://dcc.icgc.org/), and the clinical and microarray-based gene expression data from E-MTAB-6134 were downloaded from ArrayExpress (Supplementary Table 2). The basic clinical information of these data is listed in Table 3.

**Table 3:** Basic information of patient from the included datasets.

	E-MTAB-6134 (n = 288)	PACA-CA (n = 214)	PACA-AU* (n = 266)	TCGA (n = 148)	GSE85916 (n = 79)	GSE62452 (n = 65)
**Sex**						
Male	166	128	142	79	NA	NA
Female	122	86	124	69	NA	NA
Age (mean ± SD, years)	NA	65.2 ± 11.0	66.6 ± 10.6	65.0 ± 10.8	NA	NA
**Status**						
Alive	107	34	106	67	22	16
Dead	181	180	160	81	57	49
Follow-up (mean ± SD, Months)	29.8 ± 26.9	22.6 ± 20.8	18.3 ± 13.7	17.5 ± 13.9	28.0 ± 30.5	20.2 ± 16.7
**T-stage**						
T1	12	NA	NA	5	NA	NA
T2	39	NA	3	16	NA	NA
T3	237	NA	4	123	NA	NA
T4	0	NA	NA	3	NA	NA
**N-stage**						
N0	72	NA	5	39	NA	NA
N1	216	NA	1	108	NA	NA
Nx	0	NA	6	1	NA	NA
**M-stage**						
M0	NA	NA	NA	67	NA	NA
M1	NA	NA	NA	4	NA	NA
Mx	NA	NA	NA	77	NA	NA
**AJCC stage**						
I	NA	55	1	12	NA	4
II	NA	89	2	127	NA	44
III	NA	8	NA	3	NA	10
IV	NA	0	NA	4	NA	6
Uncertain	NA	62	NA	2	NA	1
**Grade**						
G1	110	NA	NA	20	NA	2
G2	130	NA	NA	84	NA	32
G3	48	NA	NA	43	NA	29
G4	0	NA	NA	1	NA	1
Gx	0	NA	NA	0	NA	1

*The downloaded data from PACA-AU on T stage, N stage and AJCC stage was not complete and the number represents the available data. *NA* Not Available.

### Identification of survival-related genes from gemcitabine resistance-related genes and establishment of the prognostic gene signature

**Figure 6:**
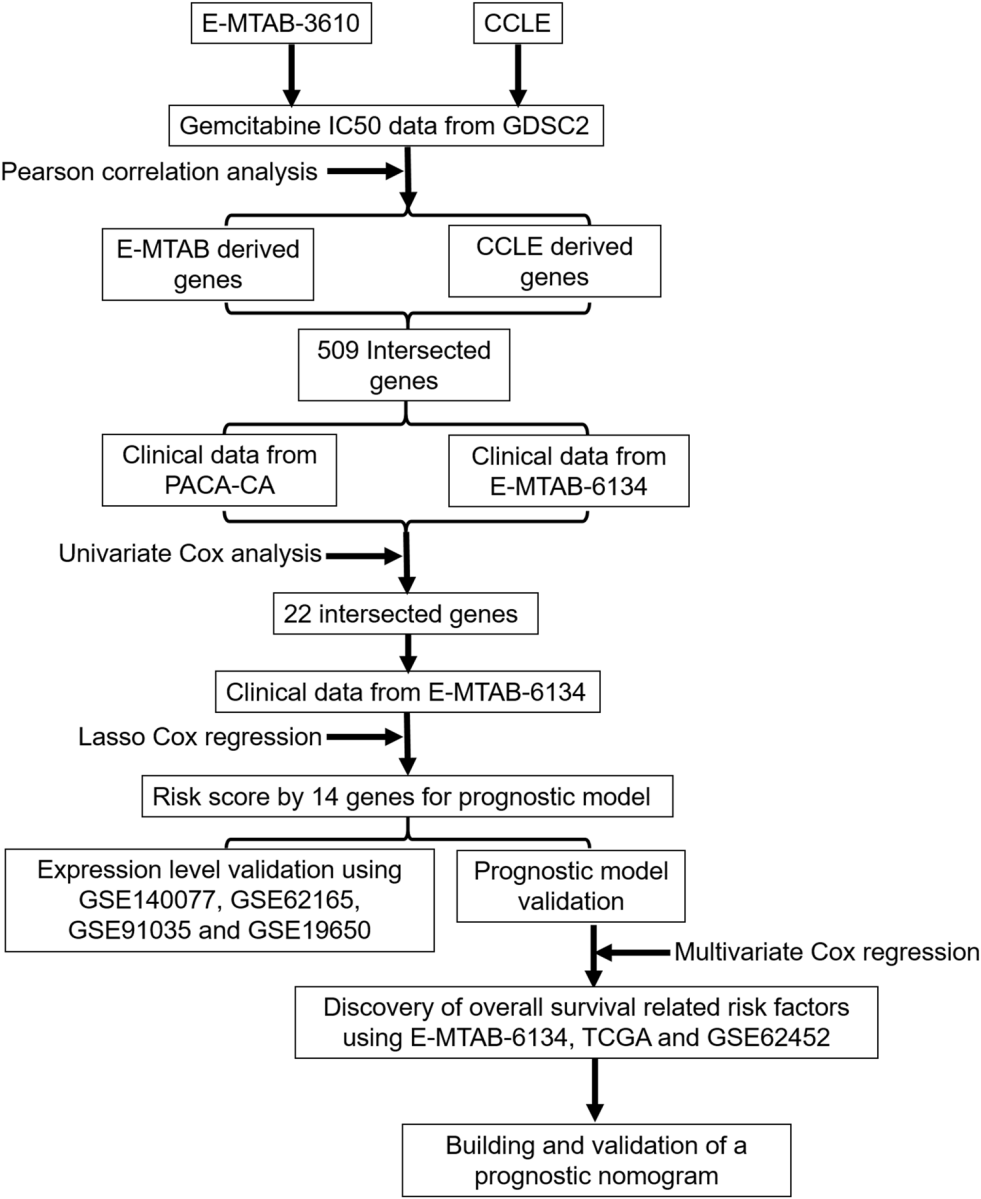
Flowchart of the data processing procedure.

Genes related to gemcitabine resistance were identified with gemcitabine IC50 data of pancreatic cancer cell lines from GDSC and the gene expression data from both the E-MTAB-3610 and CCLE datasets using Pearson correlation with the criterion of *p* < 0.05 as determined cor.test () function in the R base package. Among the cells with IC50 data, a total of 17 and 15 primary pancreatic carcinoma-derived cell lines were obtained from GDSC and CCLE, respectively, and detailed information on the cell lines used in the present study is listed in Supplementary Table 3. The intersecting genes from the above E-MTAB-3610 and CCLE datasets were then used for survival-related gene screening by the combined use of the clinical data from the E-MTAB-6134 and PACA-CA datasets by univariate Cox analysis with the criterion of *p* < 0.05. Lasso-penalized Cox regression analysis based on the glmnet package in R was performed with the intersection of the survival-related genes to further minimize the gene numbers in the selected panel while maintaining the best predictive performance using tenfold cross validation. A pancreatic cancer patient prognostic gene signature using the risk score was calculated based on a linear combination of the regression coefficients (β) from the Lasso Cox regression model multiplied by the corresponding expression level of mRNA, and an optimal 14-gene combination was obtained using Ln (lambda.min). Risk scores were divided into high- and low-risk groups based on the median risk score. The flowchart of the data processing procedure is shown in Fig. 6.

### Statistical analysis

Statistical analysis was carried out using R version 3.6.0. Student's t-test was used for between-group comparisons, while one-way ANOVA was used for multiple-group comparisons. Kaplan–Meier (KM) analysis was carried out to determine survival outcomes. The median values were used as cutoff thresholds to plot the KM curves, and the statistical significance was evaluated by the log rank test (survival and survminer R packages). The survival probability prediction for 1-, 3- and 5-year survival was calculated by receiver operator characteristic (ROC) curves and the area under the curve (AUC) (survivalROC and ROCR R packages). Univariate and multivariate Cox regression analyses with all the available clinical information were performed for survival predictive nomogram construction and validation (forestplot and rms R packages). The hazard ratio (HR) and 95% confidence interval (CI) were employed to confirm genes associated with overall survival. Unless specifically mentioned, *P* < 0.05 was considered statistically significant.

## Supplementary Information

Supplementary Information

## References

[1] Siegel RL, Miller KD, Jemal A (2020). Cancer statistics, 2020. CA Cancer J. Clin..

[2] Jia X (2018). Pancreatic cancer mortality in china: characteristics and prediction. Pancreas.

[3] Jones RP (2019). Patterns of Recurrence after resection of pancreatic ductal adenocarcinoma: a secondary analysis of the ESPAC-4 randomized adjuvant chemotherapy trial. JAMA Surg..

[4] Von Hoff DD (2013). Increased survival in pancreatic cancer with nab-paclitaxel plus gemcitabine. N. Engl. J. Med..

[5] Kim S (2018). Comparative effectiveness of nab-paclitaxel plus gemcitabine vs FOLFIRINOX in metastatic pancreatic cancer: a retrospective nationwide chart review in the United States. Adv Ther..

[6] Karasic TB (2019). Effect of gemcitabine and nab-paclitaxel with or without hydroxychloroquine on patients with advanced pancreatic cancer: a phase 2 randomized clinical trial. JAMA Oncol..

[7] Amrutkar M, Gladhaug IP (2017). Pancreatic cancer chemoresistance to gemcitabine. Cancers.

[8] de Sousa Cavalcante L, Monteiro G (2014). Gemcitabine: metabolism and molecular mechanisms of action, sensitivity and chemoresistance in pancreatic cancer. Eur. J. Pharmacol..

[9] Adamska A (2018). Molecular and cellular mechanisms of chemoresistance in pancreatic cancer. Adv. Biol. Regul..

[10] Weinstein JN (2013). The cancer genome atlas pan-cancer analysis project. Nat. Genet..

[11] Zhang J (2019). The international cancer genome consortium data portal. Nat. Biotechnol..

[12] Rustici G (2013). ArrayExpress update–trends in database growth and links to data analysis tools. Nucl. Acids Res..

[13] Yang W (2012). Genomics of Drug Sensitivity in Cancer (GDSC): a resource for therapeutic biomarker discovery in cancer cells. Nucl. Acids Res..

[14] Barretina J (2012). The Cancer Cell Line Encyclopedia enables predictive modelling of anticancer drug sensitivity. Nature.

[15] Raman P, Maddipati R, Lim KH, Tozeren A (2018). Pancreatic cancer survival analysis defines a signature that predicts outcome. PLoS ONE.

[16] Yan X (2019). Importance of gene expression signatures in pancreatic cancer prognosis and the establishment of a prediction model. Cancer Manag Res.

[17] Birnbaum DJ (2017). A 25-gene classifier predicts overall survival in resectable pancreatic cancer. BMC Med..

[18] Bailey P (2016). Genomic analyses identify molecular subtypes of pancreatic cancer. Nature.

[19] Collisson EA (2011). Subtypes of pancreatic ductal adenocarcinoma and their differing responses to therapy. Nat. Med..

[20] Moffitt RA (2015). Virtual microdissection identifies distinct tumor- and stroma-specific subtypes of pancreatic ductal adenocarcinoma. Nat. Genet..

[21] Puleo F (2018). Stratification of pancreatic ductal adenocarcinomas based on tumor and microenvironment features. Gastroenterology.

[22] Balachandran VP, Gonen M, Smith JJ, DeMatteo RP (2015). Nomograms in oncology: more than meets the eye. Lancet Oncol..

[23] Wu M, Li X, Zhang T, Liu Z, Zhao Y (2019). Identification of a nine-gene signature and establishment of a prognostic nomogram predicting overall survival of pancreatic cancer. Front Oncol..

[24] Kandimalla R (2020). A 15-gene immune, stromal and proliferation gene signature that significantly associates with poor survival in patients with pancreatic ductal adenocarcinoma. Clin. Cancer Res..

[25] Xu F (2019). Cytoplasmic PARP-1 promotes pancreatic cancer tumorigenesis and resistance. Int. J. Cancer.

[26] Sauter DRP, Novak I, Pedersen SF, Larsen EH, Hoffmann EK (2015). ANO1 (TMEM16A) in pancreatic ductal adenocarcinoma (PDAC). Pflugers Arch..

[27] Cheng Y (2019). Identification of candidate diagnostic and prognostic biomarkers for pancreatic carcinoma. EBioMedicine.

[28] Zhai S (2019). INPP4B As a prognostic and diagnostic marker regulates cell growth of pancreatic cancer via activating AKT. Oncol. Targets Ther..

[29] Kayashima T (2011). Insig2 is overexpressed in pancreatic cancer and its expression is induced by hypoxia. Cancer Sci..

[30] Smith TK (2008). Bves directly interacts with GEFT, and controls cell shape and movement through regulation of Rac1/Cdc42 activity. Proc. Natl. Acad. Sci. U S A.

[31] Yang SN, Berggren PO (2006). The role of voltage-gated calcium channels in pancreatic beta-cell physiology and pathophysiology. Endocr Rev.

[32] Pilarsky C (2008). Activation of Wnt signalling in stroma from pancreatic cancer identified by gene expression profiling. J. Cell. Mol. Med..

[33] Horpaopan S (2015). Genome-wide CNV analysis in 221 unrelated patients and targeted high-throughput sequencing reveal novel causative candidate genes for colorectal adenomatous polyposis. Int. J. Cancer.

[34] Kim TW (2014). SH3RF2 functions as an oncogene by mediating PAK4 protein stability. Carcinogenesis.

[35] Kim BK (2019). PI3K/AKT/beta-catenin signaling regulates vestigial-like 1 which predicts poor prognosis and enhances malignant phenotype in gastric cancer. Cancers.

[36] Leyden J (2006). Net1 and Myeov: computationally identified mediators of gastric cancer. Br. J. Cancer.

[37] Costantino L (2014). Break-induced replication repair of damaged forks induces genomic duplications in human cells. Science.

