# Supplementary material for: A 14-gene gemcitabine resistance gene signature is significantly associated with the prognosis of pancreatic cancer patients
Source: Sci Rep. 2021 Mar 17;11:6087. doi: 10.1038/s41598-021-85680-x (PMC7969955; doi:10.1038/s41598-021-85680-x)
Supplement: Supplementary file 1 — Supplementary Information [file 41598_2021_85680_MOESM1_ESM.pdf]

A 14-gene gemcitabine resistance gene signature is significantly associated with the prognosis of pancreatic cancer patients

Running Title:14-gene gemcitabine-resistant signature for PC

Xing Wei[1], Xiaochong Zhou[1], Yun Zhao[3,4,6], Yang He[2,4,5], Zhen Weng[2,3,4,6], Chunfang Xu[1]

1.Department of Gastroenterology, the First Affiliated Hospital of Soochow University, Suzhou 215006, China.

2.MOE Engineering Center of Hematological Disease, Soochow University, 215123, Suzhou, China.

3.Cyrus Tang Hematology Center, Soochow University, 215123, Suzhou, China.

4.National Clinical Research Center for Hematologic Diseases, the First Affiliated Hospital of Soochow University, Suzhou 215006, China.

5.MOH Key Lab of Thrombosis and Hemostasis, Jiangsu Institute of Hematology, The First Affiliated Hospital of Soochow University, Suzhou 215006, China

6.Collaborative Innovation Center of Hematology, Soochow University, 215006, Suzhou, China.

Corresponding to Zhen Weng, Ph.D., and Chunfang Xu, M.D.

E-mail: zhweng@suda.edu.cn for Z.W; xcf601@163.com for C.X.

Supplementary Figure 1. Kaplan-Meier curve, receiver operator characteristic curves for 1, 3 and 5-year overall survival prediction and 14 genes expression heatmaps of GSE71729 were shown.

Supplementary Figure 2. The detailed information for risk score and survival statue of E-MTAB-6134, PACA-CA, PACA-AU, TCGA, GSE85916, GSE62452 andGSE71729 (from top).

Supplementary Figure 3. Univariate analysis of clinical parameters and 14-gene risk score for overall survival in PACA-AU and PACA-CA.

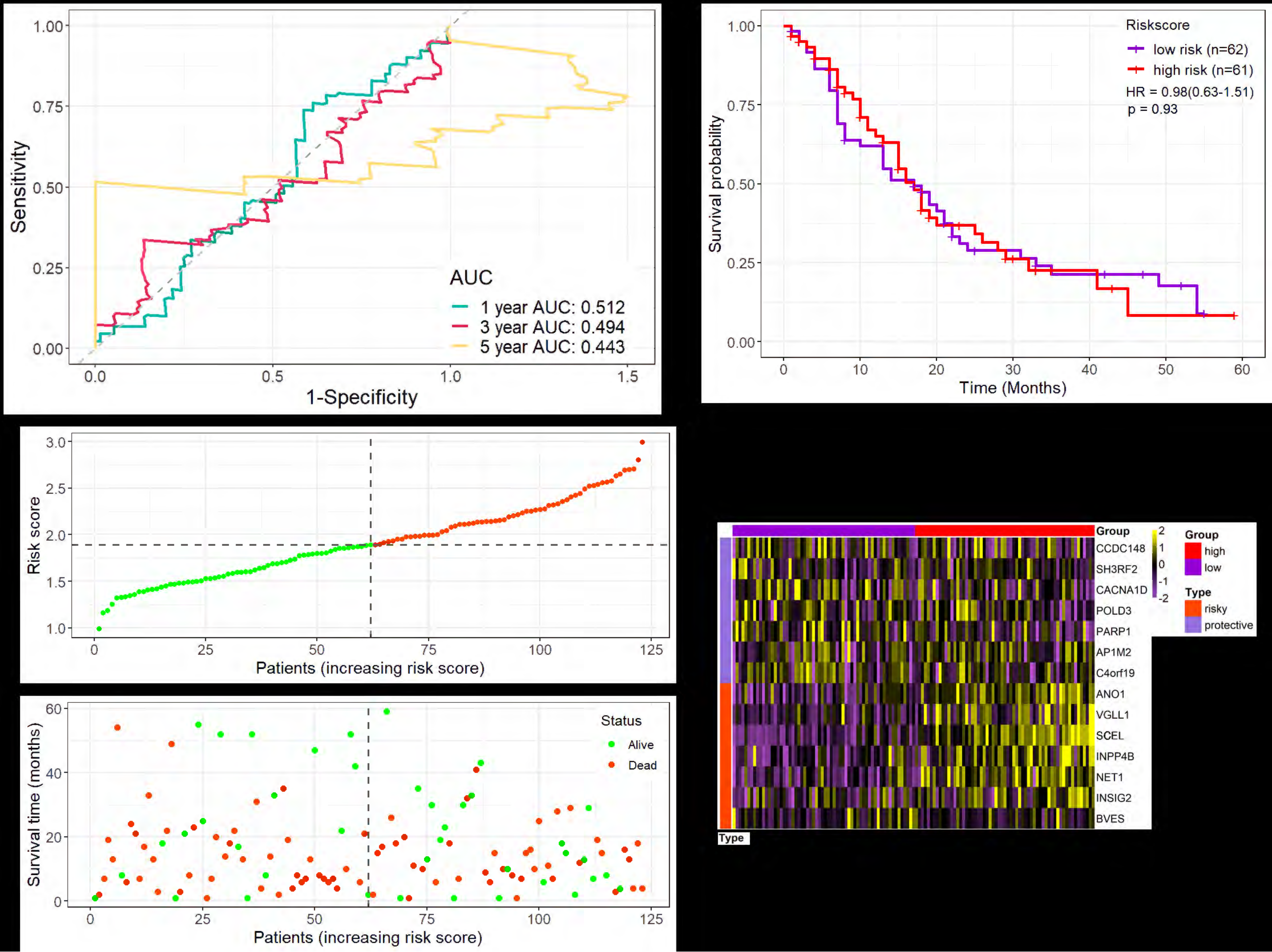

Sensitivity
1-Specificity
1.00
0.75
0.50
0.25
0.00
0.0
0.5
1.0
1.5
AUC
1 year AUC: 0.512
3 year AUC: 0.494
5 year AUC: 0.443
Riskscore
low risk (n=62)
high risk (n=61)
HR = 0.98(0.63-1.51)
p = 0.93
Survival probability
Time (Months)
0
10
20
30
40
50
60
Risk score
3.0
2.5
2.0
1.5
1.0
Patients (increasing risk score)
0
25
50
75
100
125
Survival time (months)
60
40
20
0
Status
Alive
Dead
Patients (increasing risk score)
Group
CCDC148
SH3RF2
CACNA1D
POLD3
PARP1
AP1M2
C4orf19
ANO1
VGLL1
SCEL
INPP4B
NET1
INSIG2
BVES
2
1
0
-1
-2
Group
high
low
Type
risky
protective
Type

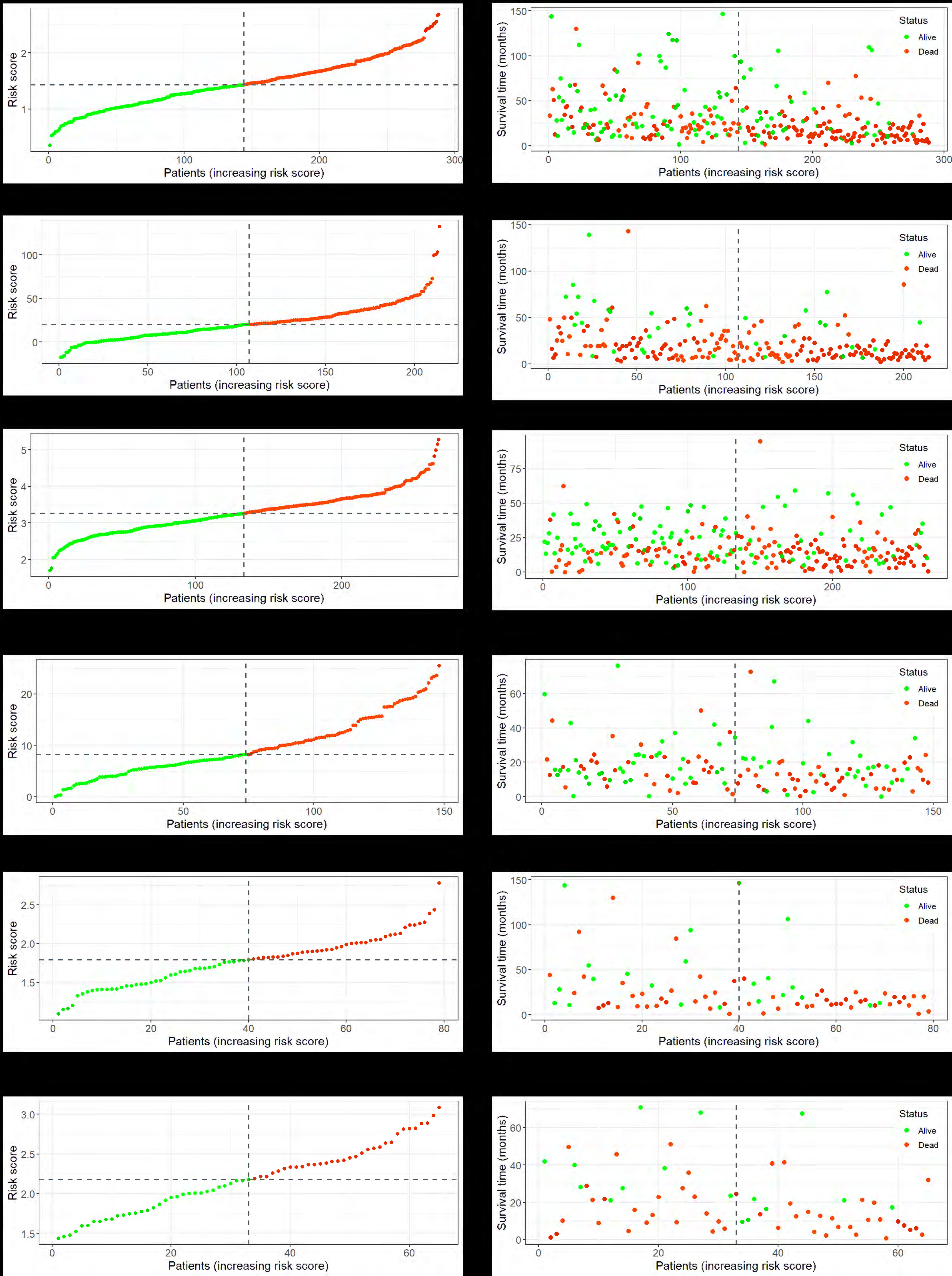

Risk score
Patients (increasing risk score)
Survival time (months)
Status
Alive
Dead

PACA-AU

	Hazard Ratio	95% CI	p-value
Gender (M vs F)	1.234	0.900-1.692	0.192
Age (≥60 vs <60)	0.938	0.647-1.361	0.737
Risk score (High vs Low)	1.899	1.380-2.614	<0.001

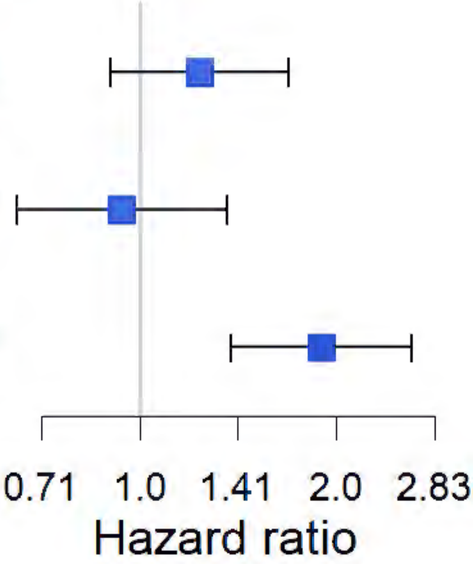

PACA-CA

	Hazard Ratio	95% CI	p-value
Gender (M vs F)	1.150	0.851-1.553	0.364
Age (≥60 vs <60)	0.856	0.603-1.215	0.384
Risk score (High vs Low)	1.761	1.307-2.371	<0.001

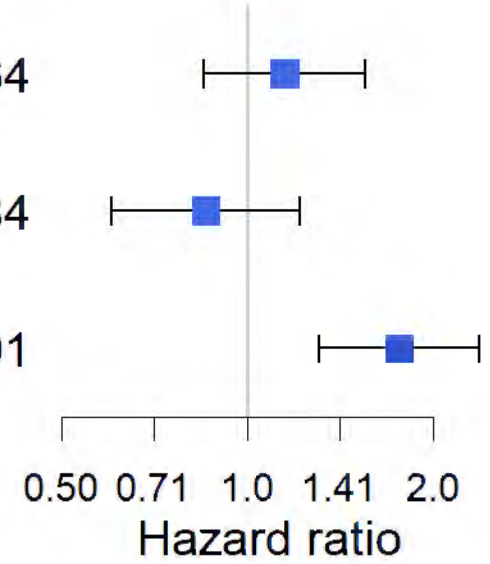

Supplementary table 1. Detailed information of the included datasets

Series	Platform	Platform Name	Sample information
**GSE140077**	GPL20795	HiSeq X Ten (Homo sapiens)	2 Gemcitabine (GEM)-resistant cell lines (BxPC-3-GR and CFPAC-1-GR) and 2 parental (BxPC-3 and CFPAC-1), each with triplicates.
**GSE19650**	GPL570	[HG-U133_Plus_2] Affymetrix Human Genome U133 Plus 2.0 Array	22 normal and neoplastic epithelial cells from frozen tissue sections (normal main pancreatic duct, IPMA, IPMC, and invasive carcinoma originating in IPMN) .
**GSE62165**	GPL13667	[HG-U219] Affymetrix Human Genome U219 Array	118 pancreatic ductal adenocarcinoma (PDAC) samples and 13 control samples.
**GSE62452**	GPL6244	[HuGene-1_0-st] Affymetrix Human Gene 1.0 ST Array [transcript (gene) version]	45 matching pairs of PDAC and adjacent non-tumor tissues
**GSE71729**	GPL20769	Agilent-014850 Whole Human Genome Microarray 4x44K G4112F (Gene Symbol Version; updated July 2014)	145 primary and 61 metastatic PDAC tumors, 17 cell lines, 46 pancreas and 88 distant site adjacent normal samples
**GSE85916**	GPL13667	[HG-U219] Affymetrix Human Genome U219 Array	118 patients with human resected pancreatic cancer
**GSE91035**	GPL22763	Agilent-039714 LincRNA SurePrint G3 Human GE 8x60K Microarray PVD 028004 [Probe Name version]	8 normal, 15 adjacent benign and 27 PDAC pancreatic tissues of human

Supplementary Table 2. Database information used

Name	Sample information	Website link
**CCLE**	RNAseq gene expression data fpr 1019 cell lines	https://portals.broadinstitute.org/ccle/data
**PACA-AU**	266 patients with both gene expression data and survival data	https://dcc.icgc.org/projects/PACA-AU
**PACA-CA**	234 patients with gene expression data and survival data	https://dcc.icgc.org/projects/PACA-CA
**TCGA Pancreatic Cancer (PAAD)**	148 patients with both gene expression data and survival data	https://xenabrowser.net/datapages/
**E-MTAB-6134**	288 patients with both expression data and survival data	https://www.ebi.ac.uk/arrayexpress/experiments/E-MTAB-6134/

Supplementary Table 3. The information for 17 primary pancreas carcinoma derived cell lines used in present study.

Name	Organism	Derived Tissue	Cell type	Growth properties	Disease	Age	Gender	Ethnicity	Source
**Capan-2**	Human	pancreas	polygonal	adherent	adenocarcinoma	56	M	Caucasian	ATCC
**Panc 08.13**	Human	pancreas	epithelial	adherent	adenocarcinoma	85	M	White	ATCC
**HPAC**	Human	pancreas	epithelial	adherent	adenocarcinoma	64	F	Caucasian	ATCC
**KP-2**	Human	pancreas	NA	NA	adenocarcinoma	65	F	NA	Pubmed
**PA-TU-8902**	Human	pancreas	epithelial	adherent	adenocarcinoma	44	F	NA	NA
**Panc 04.03**	Human	pancreas	epithelial	adherent	adenocarcinoma	70	M	White	ATCC
**HPAF-II**	Human	pancreas	epithelial	adherent	adenocarcinoma	44	M	Caucasian	ATCC
**Panc 02.03**	Human	pancreas	epithelial	adherent	adenocarcinoma	70	F	White	ATCC
**Panc 10.05**	Human	pancreas	epithelial	adherent	adenocarcinoma	NA	M	Caucasian	ATCC
**PL4**	Human	pancreas	NA	adherent	NA	NA	NA	NA	NA
**BxPC-3**	Human	pancreas	epithelial	adherent	adenocarcinoma	61	F	NA	ATCC
**MIA PaCa-2**	Human	pancreas	epithelial	adherent	carcinoma	65	M	Caucasian	ATCC
**DAN-G**	Human	pancreas	NA	NA	carcinoma	NA	NA	NA	DSMZ
**Panc 03.27**	Human	pancreas	epithelial	adherent	adenocarcinoma	65	F	White	ATCC
**KP-4**	Human	pancreas	epithelial-like	NA	carcinoma	50	M	NA	NA
**PL18**	Human	pancreas	NA	NA	adenocarcinoma	NA	NA	Asia	NA
**PSN-1**	Human	pancreas	epithelial-like	adherent	adenocarcinoma	NA	NA	NA	ATCC

F: female; M: male; NA: not available; ATCC: American Type Culture Collection; DSMZ: German Collection of Microorganisms and Cell Cultures; The unit for Age is year.
